# Programmed death‐ligand 1 gene expression is a prognostic marker in early breast cancer and provides additional prognostic value to 21‐gene and 70‐gene signatures in estrogen receptor‐positive disease

**DOI:** 10.1002/1878-0261.12654

**Published:** 2020-03-20

**Authors:** Ioannis Zerdes, Emmanouil G. Sifakis, Alexios Matikas, Sebastian Chrétien, Nicholas P. Tobin, Johan Hartman, George Z. Rassidakis, Jonas Bergh, Theodoros Foukakis

**Affiliations:** ^1^ Department of Oncology‐Pathology Karolinska Institute Stockholm Sweden; ^2^ Breast Center Theme Cancer Karolinska University Hospital Stockholm Sweden; ^3^ Department of Pathology and Cytology Karolinska University Hospital Stockholm Sweden

**Keywords:** breast cancer, gene expression, gene signatures, PD‐L1, prognosis

## Abstract

Gene and protein expression of programmed death‐ligand 1 (PD‐L1) are prognostic in early breast cancer (BC), but their prognostic information is inconsistent at least in some biological subgroups. The validated prognostic gene signatures (GS) in BC are mainly based on proliferation and estrogen receptor (ER)‐related genes. Here, we aimed to explore the prognostic capacity of PD‐L1 expression at the protein vs mRNA levels and to investigate the prognostic information that *PD‐L1* can potentially add to routinely used GS. Gene expression data were derived from two early BC cohorts (cohort 1: 562 patients; cohort 2: 1081 patients). Tissue microarrays from cohort 1 were immunohistochemically (IHC) stained for PD‐L1 using the SP263 clone. GS scores (21‐gene, 70‐gene) were calculated, and likelihood‐ratio (LR) tests and concordance indices were used to evaluate the additional prognostic information for each signature. The immune cell composition was also evaluated using the CIBERSORT *in silico* tool. PD‐L1 gene and protein expressions were independently associated with better prognosis. In ER+/HER2− patients, *PD‐L1* gene expression provided significant additional prognostic information beyond that of both 21‐GS [LR‐Δχ^2^ = 15.289 and LR‐Δχ^2^ = 8.812, *P* < 0.01 for distant metastasis‐free interval (DMFI) in cohorts 1 and 2, respectively] and 70‐GS score alone (LR‐Δχ^2^ = 18.198 and LR‐Δχ^2^ = 8.467, *P* < 0.01 for DMFI in cohorts 1 and 2, respectively). *PD‐L1* expression was correlated with IHC‐determined CD3+ cells (*r* = 0.41, *P* < 0.001) and with CD8+ (*r* = 0.62, *P* < 0.001) and CD4+ memory activated (*r* = 0.66, *P* < 0.001) but not with memory resting (*r *= −0.063, *P* = 0.14) or regulatory (*r *= −0.12, *P* < 0.01) T cells *in silico*. *PD‐L1* gene expression represents a promising favorable prognostic marker and can provide additional prognostic value to 21‐ and 70‐gene scores in ER+/HER2− BC.

## Graphical abstract

Programmed death‐ligand 1 (PD‐L1) immunohistochemical and gene expressions represent favorable prognostic factors in early breast cancer patients and were also associated with high immune infiltration. Furthermore, PD‐L1 gene expression provided significant additional prognostic information beyond that of both 21‐gene and 70‐gene signature scores alone in ER+/HER2− disease.

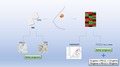

**Abbreviations**
BC: breast cancer
CI: confidence interval
CIBERSORT: cell‐type identification by estimating relative subsets of RNA transcripts
c‐index: concordance index
DMFI: distant metastasis‐free interval
ER: estrogen receptor
FISH: fluorescence *in situ* hybridization
GEP: gene expression profiling
GS: gene signatures
HER2: human epidermal growth factor receptor 2
HRs: hazard ratios
IHC: immunohistochemistry
KM: Kaplan–Meier
LN: lymph node
LR: likelihood ratio
OS: overall survival
PAM50: prediction analysis of microarray 50
PD‐L1: programmed death‐ligand 1
PFI: progression‐free interval
PH: proportional hazards
TCGA: The Cancer Genome Atlas
TILs: tumor‐infiltrating lymphocytes
TMAs: tissue microarrays
TNBC: triple‐negative breast cancer

## 1 Introduction

Continuous developments in treatment and risk stratification of early breast cancer (BC) have steadily improved survival outcomes during the past decades. However, clinicopathologic factors such as age, tumor stage, expression of estrogen receptor (ER), and human epidermal growth factor receptor 2 (HER2) do not predict the proportional risk reduction for recurrence or death conferred by adjuvant chemotherapy (CT) (Peto *et al.*, 2012). Thus, there is a clear need to identify more precise and reliable prognostic and predictive biomarkers that can be implemented in routine practice (Foukakis and Bergh). Advances in gene expression profiling (GEP) technologies have resulted in the development of gene signatures (GS) that can complement clinical decisions to predict risk of recurrence and CT benefit (Kwa *et al.*, 2017). Although prognostication through GS has been prospectively validated and is recommended by contemporary guidelines in ER‐positive, HER2‐negative (ER+/HER2−) BC (Cardoso *et al.*, 2019), there is still risk for undertreatment, especially in patients with node‐positive disease (Matikas *et al.*, 2019a).

The prognostic role of immune microenvironment in BC has been highlighted by numerous reports, demonstrating that tumor‐infiltrating lymphocytes (TILs) predict favorable disease outcome especially in the triple‐negative (TNBC) and HER2+ subtypes (Denkert *et al.*, 2018). Moreover, immune‐related GS may provide prognostic and predictive information in BC, including early and metastatic ER+ BC (Denkert *et al.*, 2015; Foukakis *et al.*, 2018; Matikas *et al.*, 2018), a tumor type traditionally considered as nonimmunogenic (Alexandrov *et al.*, 2013). Among individual protein biomarkers, programmed cell death‐ligand 1 (PD‐L1) carries prognostic value and can also select appropriate candidates for treatment with immune checkpoint blockade (Matikas *et al.*, 2019b; Schmid *et al.*, 2018). In a comprehensive pooled‐data analysis, we have shown that higher *PD‐L1* gene (*CD274*) expression is associated with improved survival, especially in basal‐like BC, whereas significant heterogeneity is noted when PD‐L1 protein expression is evaluated by immunohistochemistry (IHC) (Matikas *et al.*, 2019b). Similar results have also been reported by others (Muenst *et al.*, 2014; Sabatier *et al.*, 2015; Schalper *et al.*, 2014). Both analytical challenges due to the use of different IHC platforms, antibodies, scoring methods, and cutoffs (Hirsch *et al.*, 2017), and the complex regulation of protein expression via genetic variations, transcription factors and post‐transcriptional modifications (Zerdes *et al.*, 2018) may contribute to the observed heterogeneity.

The clinical validity and utility of *PD‐L1* gene expression remains, however, uncertain. In addition, GS that are currently available for clinical use are mostly based on proliferation and ER‐related genes. As a result, there might be room for further refinement and optimization through the addition of immune‐related genes to known GS. In this study, we aimed to investigate and compare the prognostic value of PD‐L1 gene and protein expressions, and to further explore whether the incorporation of *PD‐L1* gene expression to known GS can provide additional prognostic information.

## 2 Methods

### 2.1 Study populations

Cohort 1 was used as the discovery cohort for gene expression and for protein analysis and has been previously described in detail by Lundberg *et al. *(2017). Patients diagnosed with primary BC in Stockholm, Sweden, during 1997–2005 were retrospectively selected using the regional Cancer Registry. Patient selection was originally based on a nested case–control design, but a direct cohort design was used in the current analysis. Data on clinical and pathological tumor characteristics, survival, locoregional/systemic treatments, and follow‐up [complete to January 10, 2015, for overall survival (OS) and December 31, 2012, for distant metastasis‐free interval (DMFI)] have been collected. The clinical endpoints used in this cohort‐based study were DMFI, defined as the period of time from date of diagnosis to the first evidence of distant metastasis; and OS, defined as the period of time from date of diagnosis to death of any cause, both censored after 15 years. The reverse Kaplan–Meier (KM) estimate (Altman *et al.*, 1995) of the median DMFI and OS follow‐up was 12.4 years and 15 years, respectively. Biospecimen Reporting for Improved Study Quality criteria for this cohort have been previously published (Lundberg *et al.*, 2017). All analyses performed in cohort 1 have been approved by the ethics committee at Karolinska Institute, Stockholm, Sweden (Dnr 2006/394‐31/3, 2006/1183‐31/2, and amendments 2016/1505‐32, 2018/789‐32, 2018/790‐32), which decided that there was no need for additional written consent for each subject. The study methodologies conformed to the standards set by the Declaration of Helsinki.

Cohort 2 was used for validation of gene expression results and included patients from The Cancer Genome Atlas (TCGA) provisional dataset [originally consisting of a total of 1100 primary breast tumors with available clinicopathologic annotation and GEP (RNA‐seq) data], retrieved from cBioPortal (Cerami *et al.*, 2012; Gao *et al.*, 2013) on November 21, 2018. The clinical endpoint used was the progression‐free interval (PFI) as recommended (Liu *et al.*, 2018), defined as the time period from the date of diagnosis to the date of the first occurrence of a new tumor event, including progression of the disease, locoregional recurrence, distant metastasis, new primary tumor, or death with disease. PFI was extracted from the standardized TCGA Pan‐Cancer Clinical Data Resource dataset (Liu *et al.*, 2018). The median PFI and OS follow‐up for this cohort (reverse KM estimate) were 2.5 and 2.6 years, respectively, while both endpoints were censored after 10 years. The analyses regarding cohort 2 have been performed in publicly available data from TCGA (https://www.cancer.gov/tcga).

**Fig. 1:**
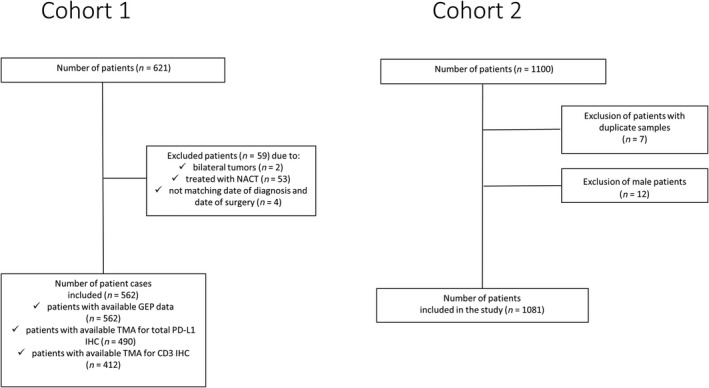
CONSORT diagrams of patient inclusion for cohorts 1 and 2.

CONSORT diagrams for both cohorts are shown in Fig. 1. This study is reported in accordance with REporting recommendations for tumor MARker prognostic studies guidelines (McShane *et al.*, 2005).

### 2.2 Tissue microarrays, immunohistochemical methods, and biomarker analysis

For cohort 1, tissue microarrays (TMAs) using primary tumors of all patients were produced. Representative tumor‐rich areas were selected and punched from formalin‐fixed paraffin‐embedded tissue blocks using an automated tissue microarrayer (VTA‐100; Veridiam, Oceanside, CA, USA). Each TMA consisted of cores with a diameter of 1 mm and duplicate cores per tumor. Tissue sections (4 μm) were prepared from the TMAs and stored at 4 °C. IHC staining with anti‐PD‐L1 (clone SP263; Ventana Medical Systems, Oro Valley, AZ, USA) and anti‐CD3 (clone 2GV6; Ventana Medical Systems) antibodies was performed using Ventana Autostainer according to the manufacturer’s protocol. PD‐L1 was evaluated separately in tumor cells and immune cells by two independent investigators including a certified pathologist. Total cell expression was defined as the expression of PD‐L1 in tumor and/or immune cells. A TMA core was considered as PD‐L1 positive (PD‐L1+) when at least one cell with membranous immunostaining was detected. Between TMA cores with discordant scoring, the positive one was selected. TMA slides stained with CD3 were scanned using a digital glass scanner (NanoZoomer‐XR, Hamamatsu Photonics K.K, Hamamatsu City, Japan), and manual scoring was performed using imagej software v. 1.48 (NIH, Bethesda, MD, USA). The total number of CD3‐positive (CD3+) cells—defined as membranous staining in lymphocytes—was counted in each TMA core and averaged over the duplicates to give the average number of CD3+ cells per tumor sample. TMA cores with missing tumor tissue were excluded from the analysis. Control tissue samples for both PD‐L1 and CD3 IHC included reactive lymphoid tissue of tonsil. ER tumor status was collected from pathology reports, while HER2 status was centrally assessed using chromogenic *in situ* hybridization on the TMAs and scored by a BC pathologist (Lundberg *et al.*, 2017).

For cohort 2, the reported ER status by IHC was used. HER2 status was determined using IHC, and for those samples with equivocal, indeterminate, or missing IHC‐based status, or for the discordant cases [defined as those cases that IHC‐based status and fluorescence *in situ* hybridization (FISH)‐based HER2 status differ], the provided FISH‐based HER2 status was used instead.

### 2.3 Gene expression profiling, data preprocessing, and normalization

In cohort 1, total RNA was extracted from primary fresh‐frozen tumors using the Qiagen RNeasy Mini Kit (Qiagen, Hilden, Germany) and samples were hybridized using the Rosetta/Merck Human RSTA Custom Affymetrix 2.0 microarray, as previously reported (Lundberg *et al.*, 2017). Further details regarding experimental methods and the microarray GEP data are available from the Gene Expression Omnibus depository (accession number http://www.ncbi.nlm.nih.gov/geo/query/acc.cgi?acc=GSE48091). The raw microarray data were background‐corrected, normalized, and summarized to obtain a log‐transformed expression value for each probe set using the RMA (Irizarry *et al.*, 2003) method implemented in the aroma.affymetrix r package (Bengtsson *et al.*, 2008). A nonspecific filter was employed, and probe sets with the highest interquartile range were kept in the case of multiple mappings to the same Entrez Gene ID.

In cohort 2, TCGA’s mRNA expression RNA sequencing data (RNA‐seq v2 RSEM) were downloaded from cbioportal (Cerami *et al.*, 2012; Gao *et al.*, 2013) on November 21, 2018. Briefly, these level 3 data have been produced after alignment of the raw reads to the human h19 genome assembly using mapsplice (Wang *et al.*, 2010), quantitation at the gene (and isoform) level using rsem (Li and Dewey, 2011), and then applying upper‐quartile normalization (Bullard *et al.*, 2010). The retrieved normalized data were log2‐transformed after addition of 1 to each value. For the analysis of *PD‐L1* mRNA levels, the median expression was used as a cutoff point in both cohorts.

### 2.4 Gene expression signatures

The same intrinsic molecular subtyping procedure was applied to both cohorts using the research‐based 50‐gene subtype predictor [prediction analysis of microarray 50 (PAM50)] (Parker *et al.*, 2009). Specifically, due to ER status imbalances, a similar to the iterative approaches (Ciriello *et al.*, 2015; Curtis *et al.*, 2012) was adopted. First, the mRNA expression data were subsampled to match the original ER distribution of the training set used for the PAM50 (Parker *et al.*, 2009), and an ER‐balanced subset was formed (using all ER− samples and randomly selected ER+ ones in a ratio of ER+/ER− = 114/77). Then, the whole dataset was median‐centered (as recommended in Perou *et al.*, 2010; Sorlie *et al.*, 2010) based on the PAM50 genes of the ER‐balanced subset, and assignment to one of the intrinsic molecular subtypes (Luminal A, Luminal B, HER2‐enriched, Basal‐like, and Normal‐like) was performed using the Spearman’s rank correlation coefficient to the PAM50 centroids available in the genefu r/bioconductor package (Gendoo *et al.*, 2016). Samples with correlations < 0.1 for all intrinsic subtypes were considered as not classified (Curtis *et al.*, 2012). The subsampling was performed 100 times, and the final intrinsic subtypes were determined by calculating the mode (i.e., the subtype that appears most often) across all iterations (Curtis *et al.*, 2012).

The original signature algorithms for 21‐gene (OncotypeDx) (Paik *et al.*, 2004) and 70‐gene (MammaPrint) (van 't Veer *et al.*, 2002) as implemented in the genefu r/bioconductor package (Gendoo *et al.*, 2016) were used to compute the corresponding research‐based signature scores and risk classifications. Before signature application, the GEP data were median‐centered. Mapping of genes for both signatures was performed through Entrez Gene IDs. Out of the 16 nonreference genes in the 21‐gene signature, in total 15 and 16 genes were available in cohorts 1 and 2, respectively, and therefore used in the signature’s calculations. Similarly, out of the 56 genes with available Entrez Gene ID in the 70‐gene signature, in total 51 and 52 genes were available in cohorts 1 and 2, respectively.

### 2.5 Quantification of immune cell subpopulations from GEP data

To deconvolve the immune cell subpopulations using the patients’ GEPs, the cell‐type identification by estimating relative subsets of RNA transcripts (CIBERSORT) method (r script version 1.04) was followed (Newman *et al.*, 2015). Specifically, CIBERSORT was employed with the gene signature matrix LM22, which contains 547 genes that distinguish 22 human hematopoietic cell phenotypes. The deconvolution was run in absolute mode; that is, for each patient, an absolute immune fraction score was estimated by the median expression level of all genes in the signature matrix divided by the median expression level of all genes in the mixture. The default number of 100 permutations was selected.

### 2.6 Statistical analyses

Survival analyses were performed with the survival r package (Therneau, 2015) using DMFI and PFI as clinical endpoints in cohorts 1 and 2, respectively. For both cohorts, OS was also used as an endpoint. Specifically, univariate and multivariable Cox proportional hazards (PH) regression models were applied and hazard ratios (HRs) and associated 95% confidence intervals (CIs) were estimated. The PH assumption was tested for all variables using the scaled Schoenfeld residuals. For comparability, the same Cox models were applied to cohorts 1 and 2, based on a set of common covariates that were available in both cohorts. Therefore, clinical variables included as covariates were lymph node status (categorical, LN−; LN+) and tumor size (categorical, ≤ 20 mm; > 20 mm). PAM50 subtype was included as a stratifying factor in the models fitted for all patients. *PD‐L1* transcript was evaluated as continuous variable. The 21‐gene and 70‐GS were evaluated as either continuous or categorical variables. Exploratory interaction tests between the *PD‐L1* transcript expression and the predefined subgroups (either clinical or PAM50‐based) with respect to survival outcome were evaluated in multivariable models. Survival distribution differences were also assessed using KM estimates and the log‐rank test, where *PD‐L1* transcript expression was dichotomized using median as cutoff within each subtype analysis.

The added prognostic value of *PD‐L1* gene to each GS score was assessed by two approaches: (a) the changes in the likelihood‐ratio test values (LR‐Δχ^2^) and (b) the concordance index (c‐index) (Dowsett *et al.*, 2013). Each GS score was added either alone or in combination with *PD‐L1* transcript expression to a Cox PH model with the corresponding clinical endpoint(s) for each cohort.

The chi‐squared (χ^2^) test was used to assess any differences in clinicopathological characteristics between patients with positive/negative (protein) or high/low (mRNA) PD‐L1 expression and with positive/negative CD3 protein expression. Associations between transcript and protein expression levels were estimated using the Wilcoxon–Mann–Whitney test, while correlations between *PD‐L1* mRNA levels and absolute fraction scores of immune cell subpopulations were accessed using Spearman’s rank correlation coefficient. All statistical tests applied were two‐sided, and a *P*‐value < 0.05 was considered as statistically significant.

All bioinformatics and statistical analyses were performed within r computing environment version 3.5.1 (https://www.r-project.org/), unless otherwise stated.

## 3 Results

### 3.1 Patient characteristics

The clinicopathologic characteristics of patients with available GEP and IHC data in cohort 1 are summarized in Table 1 and Table S1, respectively. In brief, among 562 early BC patients with available GEP data included in the analysis, median age was 55 years (range: 23–76 years), while 312 (55.5%) patients were presented with axillary LN involvement. Tumor size was > 20mm in 288 (51.2%) patients, whereas ER, progesterone receptor (PR), and HER2 were positive in 397 (70.6%), 268 (47.7%), and 96 (17.1%) patients, respectively. In cohort 2, among 1081 patients included in the analysis, median age was 58 years (range: 26–90 years) and 761 (70.4%) had a tumor size of > 20mm. ER, PR, and HER2 were positive in 794 (73.5%), 688 (63.6%), and 179 (16.6%) patients, respectively (Table S2).

**Table 1:** Patient characteristics for all patients in cohort 1, split by *PD‐L1* mRNA (median) expression. ET, endocrine treatment; CT, chemotherapy.

Clinical and pathological characteristics	All *n* (%)	PD‐L1 mRNA low *n* (%)^a^	PD‐L1 mRNA high *n* (%)^a^	*P*‐value
Number of patients	562	281	281	
PD‐L1 protein expression (total)				
Negative	369 (65.7)	219 (59.3)	150 (40.7)	**< 0.0001**
Positive	121 (21.5)	23 (19.0)	98 (81.0)	
Unknown	72 (12.8)	39 (54.2)	33 (45.8)	
ER status				
ER−	152 (27.0)	49 (32.2)	103 (67.8)	**< 0.0001**
ER+	397 (70.6)	225 (56.7)	172 (43.3)	
Unknown	13 (2.3)	7 (53.8)	6 (46.2)	
PR status				
PR−	152 (27.0)	51 (33.6)	101 (66.4)	**< 0.0001**
PR+	268 (47.7)	157 (58.6)	111 (41.4)	
Unknown	142 (25.3)	73 (51.4)	69 (48.6)	
HER2 status				
HER2−	385 (68.5)	198 (51.4)	187 (48.6)	0.058
HER2+	96 (17.1)	39 (40.6)	57 (59.4)	
Unknown	81 (14.4)	44 (54.3)	37 (45.7)	
Elston–Ellis grade				
Grade I	46 (8.2)	30 (65.2)	16 (34.8)	**< 0.0001**
Grade II	243 (43.2)	140 (57.6)	103 (42.4)	
Grade III	244 (43.4)	95 (38.9)	149 (61.1)	
Unknown	29 (5.2)	16 (55.2)	13 (44.8)	
LN status				
Negative	233 (41.5)	111 (47.6)	122 (52.4)	0.322
Positive	312 (55.5)	162 (51.9)	150 (48.1)	
Unknown	17 (3.0)	8 (47.1)	9 (52.9)	
Tumor size				
≤ 20 mm	262 (46.6)	132 (50.4)	130 (49.6)	0.801
> 20 mm	288 (51.2)	142 (49.3)	146 (50.7)	
Unknown	12 (2.1)	7 (58.3)	5 (41.7)	
Age				
≤ 45	128 (22.8)	61 (47.7)	67 (52.3)	0.438
45–55	141 (25.1)	77 (54.6)	64 (45.4)	
> 55	293 (52.1)	143 (48.8)	150 (51.2)	
IHC subtypes				
ER+/HER2−	283 (50.4)	163 (57.6)	120 (42.4)	**< 0.0001**
HER2+	96 (17.1)	39 (40.6)	57 (59.4)	
ER−/HER2−	93 (16.5)	29 (31.2)	64 (68.8)	
Unknown	90 (16.0)	50 (55.6)	40 (44.4)	
PAM50‐based subtypes				
Luminal A	249 (44.3)	154 (61.8)	95 (38.2)	**< 0.0001**
Luminal B	107 (19.0)	61 (57.0)	46 (43.0)	
HER2‐enriched	64 (11.4)	17 (26.6)	47 (73.4)	
Basal‐like	122 (21.7)	41 (33.6)	81 (66.4)	
Normal‐like	18 (3.2)	7 (38.9)	11 (61.1)	
Unknown	2 (0.4)	1 (50.0)	1 (50.0)	
Treatment				
ET	167 (29.7)	100 (59.9)	67 (40.1)	**< 0.0001**
CT	156 (27.8)	50 (32.1)	106 (67.9)	
ET/CT	222 (39.5)	122 (55.0)	100 (45.0)	
Other treatment	16 (2.8)	9 (56.3)	7 (43.8)	
Unknown	1 (0.2)	0 (0.0)	1 (100.0)	

*P*‐values under the threshold of statistical significance are noted in bold.

^a^ Percentage (%) is calculated according to *PD‐L1* mRNA expression (low vs high group).

### 3.2 PD‐L1 mRNA and protein expression and association with clinicopathologic parameters

**Fig. 2:**
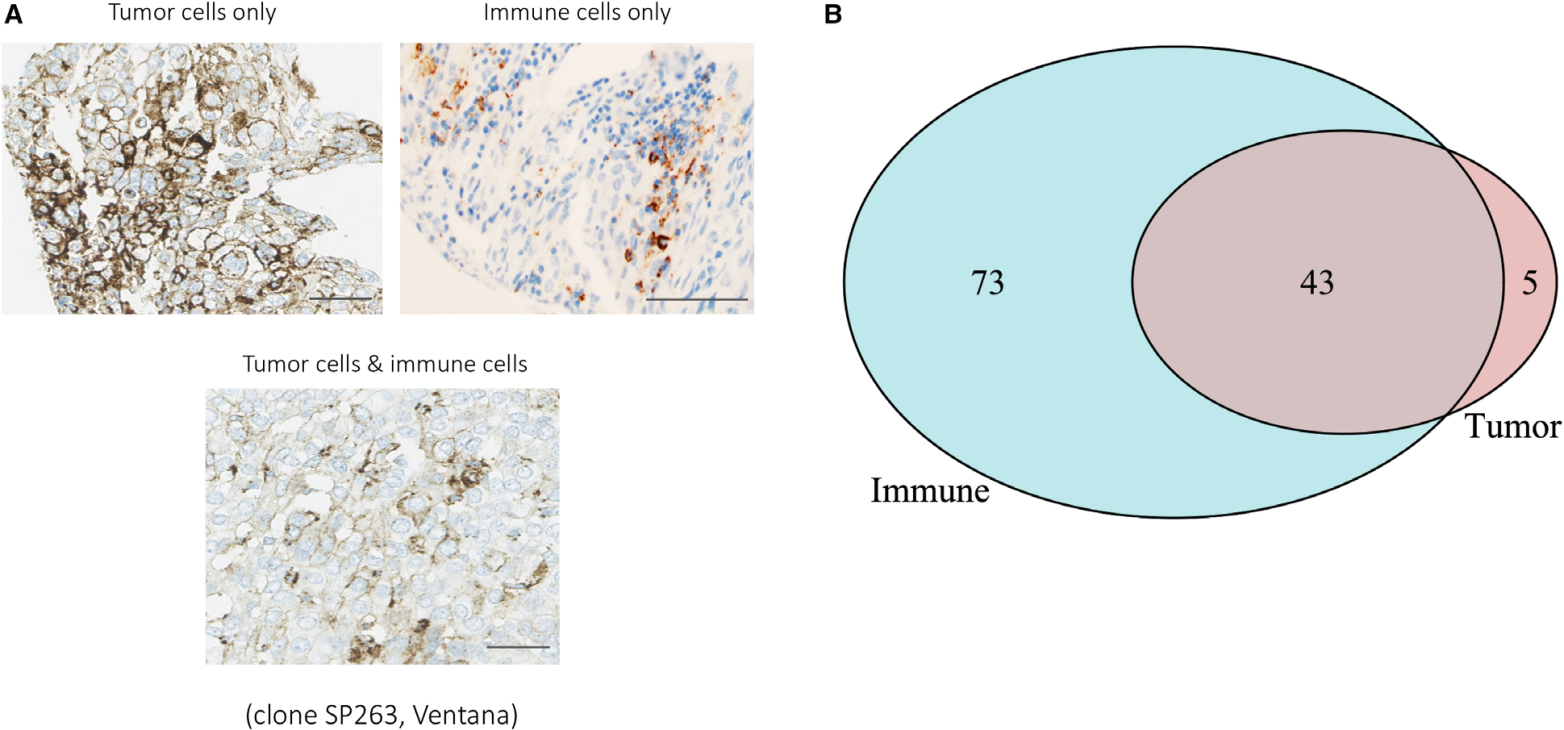
PD‐L1 protein expression patterns in BC cohort 1. (A) Areas of PD‐L1 protein expression by IHC in different cell compartments: tumor cells (upper left panel, original magnification ×400), immune cells (upper right panel, original magnification ×200), and both cell types (lower panel, original magnification ×400), scale bar 200μm; (B) Venn diagram depicting the distribution of PD‐L1 protein expression in tumor and immune cells.

Programmed death‐ligand 1 protein IHC expression was evaluable in 490 of 562 (87.2%) patients in cohort 1. PD‐L1 was expressed in tumor cells, immune cells (i.e., macrophages, dendritic cells), or both (total expression) in 9.8%, 23.7%, and 24.7% of the tumors, respectively. In the majority of patient samples with PD‐L1 expression in tumor cells, co‐expression was also noted in immune cells (Fig. 2). Within immunohistochemical and molecular subtypes, PD‐L1 total protein was highly expressed among ER−/HER2− (38/86, 44.2%) and basal‐like (48/107, 44.9%) tumors, respectively (Table S1). *PD‐L1* gene expression was higher in ER−/HER2− and molecularly basal‐like tumors in both cohorts (Table 1 and Table S2).

### 3.3 Prognostic value of PD‐L1 expression at the protein and mRNA levels

The correlation between *PD‐L1* mRNA and protein levels, as well as their prognostic value, was explored in cohort 1. *PD‐L1* transcript levels were statistically significantly correlated with protein expression (Wilcoxon, *P* < 0.001; Fig. S1). Total PD‐L1 protein expression was significantly associated with improved DMFI (univariate HR = 0.58; 95% CI = 0.39–0.87, *P* < 0.01; and multivariable HR = 0.52; 95% CI = 0.34–0.80, *P* < 0.01) and OS (univariate HR = 0.75; 95% CI = 0.53–1.05, *P* = 0.089; and multivariable HR = 0.66; 95% CI = 0.46–0.94, *P* < 0.05; Tables S3 and S4). Similar results were observed when PD‐L1 protein expression was assessed only in tumor or immune cells (Fig. S1).

Furthermore, *PD‐L1* gene expression was independently associated with better DMFI and OS in multivariable analysis (HR = 0.71; 95% CI = 0.61–0.82, *P* < 0.001; and HR = 0.77; 95% CI = 0.67–0.87, *P* < 0.001, respectively) in cohort 1 (Fig. S2 and Table S5). In cohort 2, *PD‐L1* gene expression was significantly associated with longer PFI (HR = 0.76; 95% CI = 0.64–0.91, *P* < 0.01) but not OS (HR = 0.85; 95% CI = 0.72–1.01, *P* = 0.065; Table S5). The favorable prognostic value of *PD‐L1* gene expression was more pronounced in basal‐like subtype in both cohorts and all endpoints (Fig. S2 and Table S5). In ER+/HER2− patients, *PD‐L1* mRNA was associated with better DMFI and PFI in cohorts 1 (HR = 0.71; 95% CI = 0.57–0.87, *P* < 0.01) and 2 (HR = 0.67; 95% CI = 0.50–0.89, *P* = 0.01), respectively, but with improved OS only in cohort 1 (Fig. S2 and Table S5), presumably due to the short follow‐up for OS in cohort 2.

### 3.4 Association of the immune infiltrate with PD‐L1 expression

**Fig. 3:**
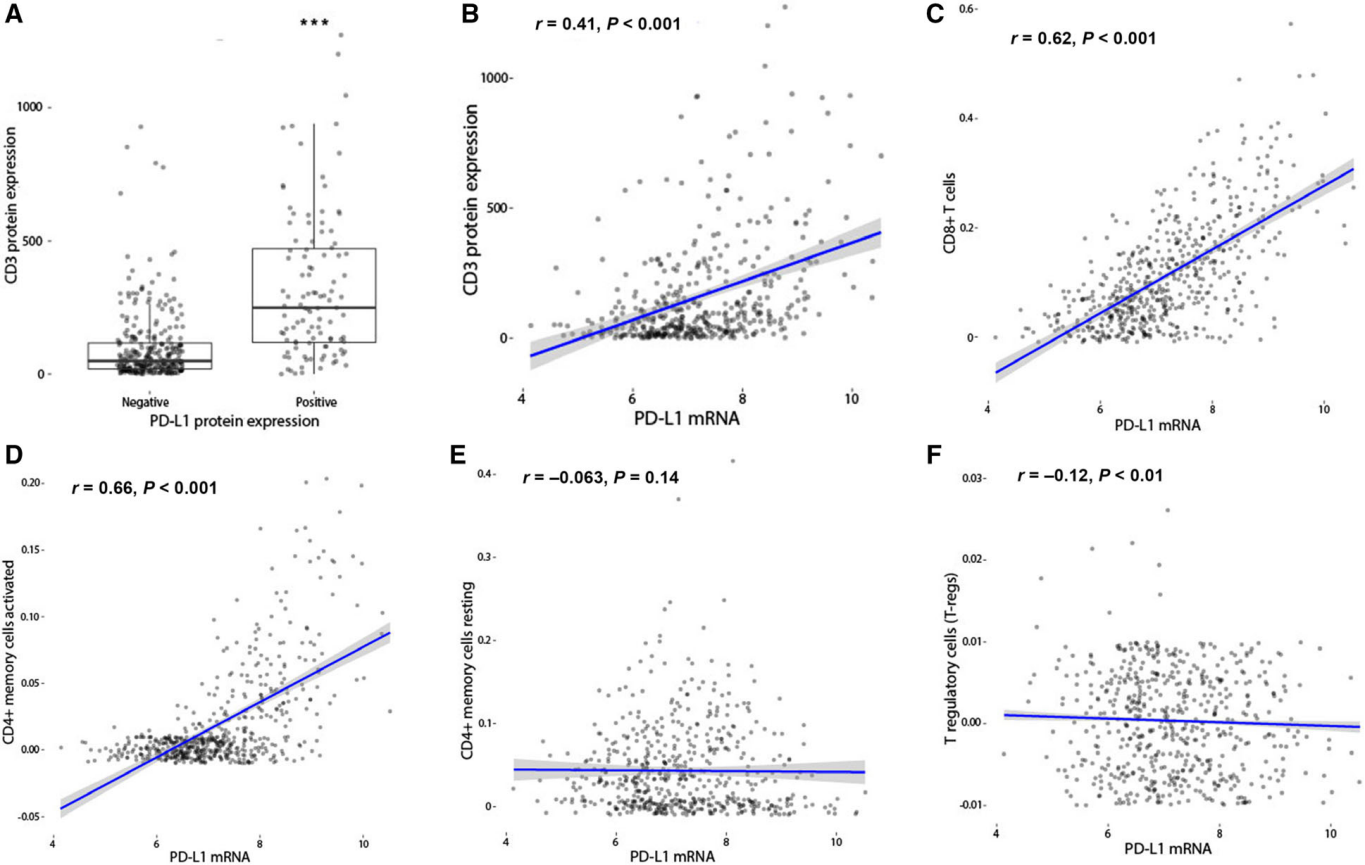
PD‐L1 expression in association with lymphocytic infiltration using IHC and *in silico* methods. Associations between CD3 IHC expression and PD‐L1 total cell protein (A) and mRNA expression (Wilcoxon–Mann–Whitney test) (B). Correlations between *PD‐L1* mRNA expression and immune cell subpopulations, as derived from the CIBERSORT *in silico* approach, CD8+ T cells (C), CD4+ memory activated T cells (D), CD4+memory resting T cells (E), and T‐regulatory cells (F) (Spearman’s rank correlation coefficient); ***P < 0.001.

T‐cell infiltration was also immunohistochemically evaluated in 412 of 562 (73.3%) patient tumors with available data in cohort 1 (Fig. 1). CD3 IHC expression was higher in triple‐negative and in basal‐like subtypes (Table S6) and predicted improved DMFI (Fig. S3) for all patients. CD3 IHC expression also significantly correlated with both PD‐L1 protein (Wilcoxon, *P* < 0.001) and mRNA (Spearman’s Rho = 0.41, *P* < 0.001) expressions (Fig. 3A,B). Further associations of *PD‐L1* expression with distinct immune cell subpopulations were explored using the CIBERSORT *in silico* approach. *PD‐L1* gene expression was positively associated with CD8+ and CD4+ memory activated T cells (Fig. 3C,D), but not with CD4+ memory resting or T‐regulatory cells (Fig. 3E,F) or other immune cell subpopulations (Fig. [Link], [Link], [Link]).

### 3.5 Additional prognostic value of *PD‐L1* gene expression to 21‐gene and 70‐gene signatures in ER+/HER2− patients

Having demonstrated the prognostic value of *PD‐L1* mRNA in ER+/HER2− patients, we aimed to investigate the additional prognostic value that it may provide to clinically used GS. When added to the 21‐gene signature, *PD‐L1* provided significant prognostic information beyond that of the GS alone in terms of DMFI/PFI (LR‐Δχ^2^ = 15.289, *P* < 0.001; and LR‐Δχ^2^ = 8.812, *P* = 0.003 in cohorts 1 and 2, respectively) and OS (LR‐Δχ^2^ = 10.020, *P* = 0.002 in cohort 1). Similarly, when added to the 70‐gene signature, *PD‐L1* provided significant prognostic information beyond that of the GS score alone in terms of DMFI/PFI (LR‐Δχ^2^ = 18.198, *P* < 0.001; and LR‐Δχ^2^ = 8.467, *P* = 0.004 in cohorts 1 and 2, respectively) and OS (LR‐Δχ^2^ = 12.468, *P* < 0.001 in cohort 1). Moreover, c‐indices were higher for RS + *PD‐L1* compared to RS alone both for DMFI and PFI (0.670 vs 0.636 and 0.666 vs 0.603 in cohorts 1 and 2, respectively) and for OS (0.624 vs 0.594 and 0.567 vs 0.565 in cohorts 1 and 2, respectively). A similar pattern was noted for the 70‐gene signature + *PD‐L1* vs the GS alone for DMFI and PFI (0.648 vs 0.607 and 0.659 vs 0.593 in cohorts 1 and 2, respectively) and for OS only in cohort 1 (c‐index: 0.612 vs 0.578; Table 2). The results were similar when GS scores were treated as categorical instead of continuous variables (Table S7).

**Table 2:** Added prognostic information of *PD‐L1* gene expression to known gene expression signatures (evaluated as continuous variables) in ER+/HER2− patients in cohorts 1 and 2.

	Cohort 1 (*n* = 283)				Cohort 2 (*n* = 590)			
	LR‐Δχ^2^	*P*‐value	LR‐Δχ^2^	*P*‐value	LR‐Δχ^2^	*P*‐value	LR‐Δχ^2^	*P*‐value
	DMFI		OS		PFI		OS	
21‐gene								
21‐gene + *PD‐L1* vs 21‐gene	15.289	< 0.001	10.020	0.002	8.812	0.003	0.038	0.845
70‐gene								
70‐gene + *PD‐L1* vs 70‐gene	18.198	< 0.001	12.468	< 0.001	8.467	0.004	0.034	0.855

	C‐index			
	DMFI	OS	PFI	OS
21‐gene				
21‐gene	0.636	0.594	0.603	0.565
21‐gene + *PD‐L1*	0.670	0.624	0.666	0.567
70‐gene				
70‐gene	0.607	0.578	0.593	0.584
70‐gene + *PD‐L1*	0.648	0.612	0.659	0.579

## 4 Discussion

During the rapidly evolving era of immunotherapy, PD‐L1 protein expression is widely used as biomarker for selection of appropriate candidates for immune checkpoint blockade in several tumor types (Reck *et al.*, 2016; Reck *et al.*, 2019), including TNBC (Emens *et al.*, 2018; Schmid *et al.*, 2018). Regarding its prognostic value, we have recently shown that PD‐L1 expression in tumor cells is associated with worse prognosis, while it is correlated with improved outcomes when expressed in immune cells in the TNBC subtype (Matikas *et al.*, 2019b). In this study, we demonstrate that PD‐L1 expression in tumor and/or immune cells is associated with improved survival outcomes, further indicating that its prognostic capacity is predominantly assay‐dependent. This finding underscores both the need for the establishment of standardized PD‐L1 IHC platform and evaluation guidelines and for further insights on the immune microenvironment biology. The latter might be of importance given that PD‐L1 expression appears here as a marker of immune cell accumulation rather than that of immune exhaustion (Hegde and Chen, 2020), as shown in our cohort both immunohistochemically and *in silico*.

The aforementioned shortcomings of PD‐L1 IHC expression pave the way for alternative approaches, such as assessing PD‐L1 expression at the mRNA level (yet not feasible in most diagnostic labs). We have previously shown that *PD‐L1* gene expression is associated with prolonged DFS and OS (Matikas *et al.*, 2019b). In the present study, we confirm that *PD‐L1* gene expression is correlated with improved outcomes, while we also demonstrate a significant correlation between PD‐L1 mRNA and protein expression, helping to clarify currently available inconsistent studies that have reported high (Guo *et al.*, 2016; Kim *et al.*, 2017), moderate, or low protein–mRNA correlation (Ali *et al.*, 2015; Ren *et al.*, 2018). *PD‐L1* mRNA can thus be a promising and reliable prognostic marker compared with current IHC methods but with unclear—up until now—clinical validity and utility.

Guidelines and indications for the usage of genomic risk prediction include a number of GS (Harris *et al.*, 2016), but only RS and 70‐gene signature have been prospectively validated in randomized trials (Cardoso *et al.*, 2016; Sparano *et al.*, 2018). Through their use, patients with ER+/HER2− BC (especially those with node‐negative disease) at sufficiently low risk of relapse can be identified, so that adjuvant CT can be omitted. Further optimization through the addition of components such as immune response might be possible, especially when such factors can carry independent prognostic and predictive information. Here, we demonstrate the clinical validity of immune gene expression, since adding *PD‐L1* to two GS improved their prognostic capacity. The improved prognostication could be due to the addition of purely prognostic information through the identification of patients at very low risk of relapse. However, we (Foukakis *et al.*, 2018; Matikas *et al.*, 2018) and others (Denkert *et al.*, 2018) have shown that immune function, expressed as immune‐related gene expression or abundance of TILs, is also a driver for chemosensitivity in ER+/HER2− BC, implying that the results of the present study might be due to increased efficacy of CT in high PD‐L1 expressors. However, since allocation to CT was not randomized, this hypothesis cannot be proven in our study. The possible clinical implications are therefore obvious, since optimization of GS currently in use may pave the way for CT de‐escalation and avoidance of unnecessary treatment‐related short‐ and long‐term adverse events.

However, this study suffers from some limitations needed to be addressed. First, PD‐L1 IHC expression was evaluated in TMAs (with duplicate cores from each tumor) rather than in whole‐tissue sections. Previous studies in BC showed that TMA protein assessment underestimated PD‐L1 expression due to its spatial heterogeneity as compared to whole‐tissue sections (Sobral‐Leite *et al.*, 2018). Moreover, SP263 clone was used for PD‐L1 IHC, which should be put into the context of low reported analytical concordance among different antibodies in BC (Rugo *et al.*, 2019). Furthermore, the studied cohorts included patients irrespective of their nodal status, thus hindering the translational interpretation of our findings specifically in node‐negative or node‐positive patients. In both cohorts (Table 1 and Table S2), patients with high‐risk characteristics were overrepresented compared with BC population. Due to the low number of time‐to‐endpoint events, no separate analysis could be performed according to the given treatment (CT vs no‐CT), and therefore, the possible predictive value of the GS and *PD‐L1* gene expression cannot be explored within these cohorts. Moreover, the GS used in this study do not represent the commercial versions of the tests and the lower percentage of available genes—especially in the 70‐gene signature—may impact its prognostic performance. Finally, the retrospective nature of the study might introduce bias, underscoring the need for prospective validation of our findings.

## 5 Conclusions

In conclusion, this study highlights the value of *PD‐L1* gene expression as an informative biomarker of good prognosis in early BC. Especially in ER+/HER2− disease, it can provide added prognostic value beyond that of 21‐ and 70‐GS. Therefore, upon method standardization and prospective validation, *PD‐L1* mRNA might be considered as a candidate biomarker for implementation in routine clinical practice.

## Conflict of interest

Ioannis Zerdes, Emmanouil Sifakis, Alexios Matikas, Sebastian Chrétien, Nicholas P. Tobin, and George Z. Rassidakis have no conflicts of interest to disclose. Johan Hartman received speakers’ honorarium and travel support from Roche, Novartis, AstraZeneca, and MSD, and institutional research support from Cepheid. Theodoros Foukakis received institutional grants from Roche and Pfizer and personal fees from Novartis, Pfizer, Roche, and UpToDate. Jonas Bergh reports that his institution (Karolinska Institute and/or Karolinska University Hospital) has received commercial research grants from AstraZeneca, Amgen, Bayer, Roche, Merck, Pfizer, and Sanofi‐Aventis, no personal payments, and payment from UpToDate for a chapter in breast cancer prediction paid to Asklepios Medicine HB.

## Author contributions

IZ, AM, and TF conceptualized and designed the study. IZ, EGS, SC, and GZR performed data acquisition and analyzed the data. All authors interpreted the data and contributed to the manuscript drafting, revision, and approval.

## Supporting information


**Fig. S1.** Prognostic value of PD‐L1 protein expression and correlation with mRNA levels according to cell of origin in cohort 1. Correlation of *PD‐L1* gene expression with protein levels in tumor (A), immune (B) and total (C) cells, (Mann‐Whitney test, ***P < 0.001); Survival analysis (Kaplan‐Meier estimate) with the DMFI as a clinical endpoint in breast cancer patients spit by PD‐L1 IHC expression in tumor (D) and immune (E) cells.

**Fig. S2.** Prognostic value of *PD‐L1* mRNA expression in cohort 1. Forest plots of HR for DMFI (A), and OS (B) both in the whole population and within clinical and PAM50‐based subtypes; HR is the relative hazard for a one‐standard deviation increase in the *PD‐L1* mRNA expression. Cox regression multivariable models were adjusted for LN status and tumor size.

**Fig. S3.** Lymphocytic infiltration and its prognostic value in cohort 1. (A) Representative tissue areas immunohistochemically (IHC) stained for CD3 with low and high lymphocytic infiltration. Original magnification x400; (B) Survival analysis (Kaplan‐Meier estimate) with the DMFI as a clinical endpoint in breast cancer patients spit by CD3 IHC expression.

**Fig. S4.** Correlation matrix depicting PD‐L1 transcript with different absolute fraction scores as derived from CIBERSORT‐based immune cell subpopulations in cohort 1.

**Table S1.** Patient characteristics for all patients and split by PD‐L1 total protein expression in cohort 1.
**Table S2.** Patient characteristics for all patients and split by *PD‐L1* mRNA (median) expression in cohort 2.
**Table S3.** Univariate analysis of PD‐L1 IHC expression with survival outcomes in cohort 1.
**Table S4.** Multivariable analysis of PD‐L1 IHC expression with survival outcomes in cohort 1.
**Table S5.** Univariate and multivariable analyses of *PD‐L1* mRNA expression with survival outcomes in both cohorts.
**Table S6.** Patient characteristics for patients split by CD3 protein expression (median) in cohort 1.
**Table S7.** Added prognostic value of *PD‐L1* mRNA to GS (categorical) in ER+/HER2− patients in both cohorts.

## Data Availability

Cohort 1: Further details regarding experimental methods and the microarray GEP data are available from the Gene Expression Omnibus depository (accession number http://www.ncbi.nlm.nih.gov/geo/query/acc.cgi?acc=GSE48091). Cohort 2: The results published here are in part based upon data generated by the TCGA Research Network: https://www.cancer.gov/tcga.
