# Supplementary material for: Programmed death‐ligand 1 gene expression is a prognostic marker in early breast cancer and provides additional prognostic value to 21‐gene and 70‐gene signatures in estrogen receptor‐positive disease
Source: Mol Oncol. 2020 Mar 20;14(5):951–63. doi: 10.1002/1878-0261.12654 (PMC7191187; doi:10.1002/1878-0261.12654)

Supplementary Figure S1. Prognostic value of PD-L1 protein expression and correlation with mRNA levels according to cell of origin in cohort 1. Correlation of PD-L1 gene expression with protein levels in tumor (A), immune (B) and total (C) cells, (Mann-Whitney test, ***, p<0.001); Survival analysis (Kaplan-Meier estimate) with the distant metastasis-free interval (DFMI) as a clinical endpoint in breast cancer patients spit by PD-L1 IHC expression in tumor (D) and immune (E) cells.

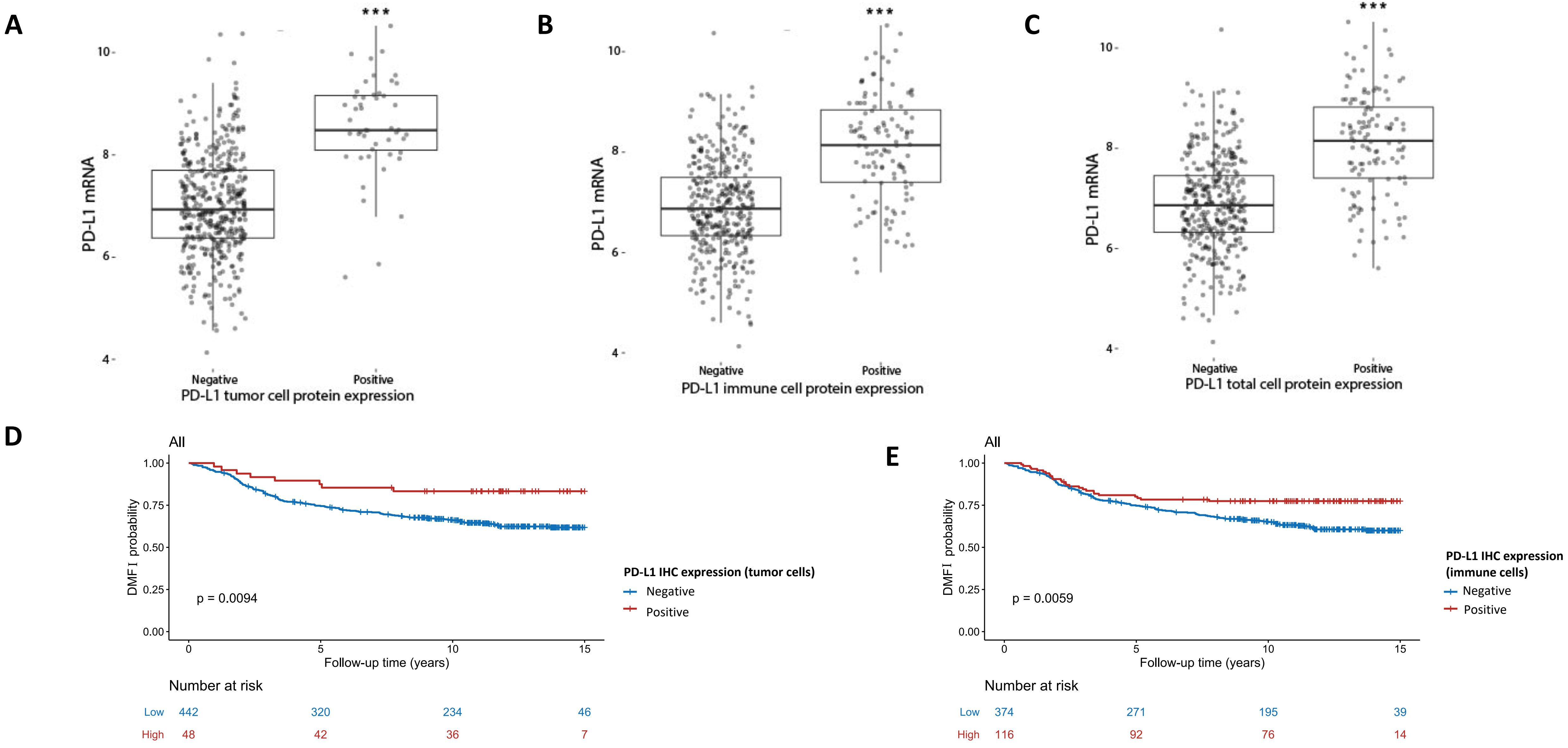

Supplement: Supplementary file 1 — Fig. S1. Prognostic value of PD‐L1 protein expression and correlation with mRNA levels according to cell of origin in cohort 1. Correlation of PD‐L1 gene expression with protein levels in tumor (A), immune (B) and total (C) cells, (Mann‐Whitney test, ***P < 0.001); Survival analysis (Kaplan‐Meier estimate) with the DMFI as a clinical endpoint in breast cancer patients spit by PD‐L1 IHC expression in tumor (D) and immune (E) cells. [file MOL2-14-951-s001.pdf]
