# Supplementary material for: Programmed death‐ligand 1 gene expression is a prognostic marker in early breast cancer and provides additional prognostic value to 21‐gene and 70‐gene signatures in estrogen receptor‐positive disease
Source: Mol Oncol. 2020 Mar 20;14(5):951–63. doi: 10.1002/1878-0261.12654 (PMC7191187; doi:10.1002/1878-0261.12654)

Supplementary Figure S2. **Prognostic value of PD-L1 mRNA expression in cohort 1. Forest plots of hazard ratios (HR) for distant metastasis-free interval (A), and overall survival (B) both in the whole population and within clinical and PAM50-based subtypes; HR is the relative hazard for a one-standard deviation increase in the PD-L1 mRNA expression. Cox regression multivariable models were adjusted for lymph node status and tumor size**

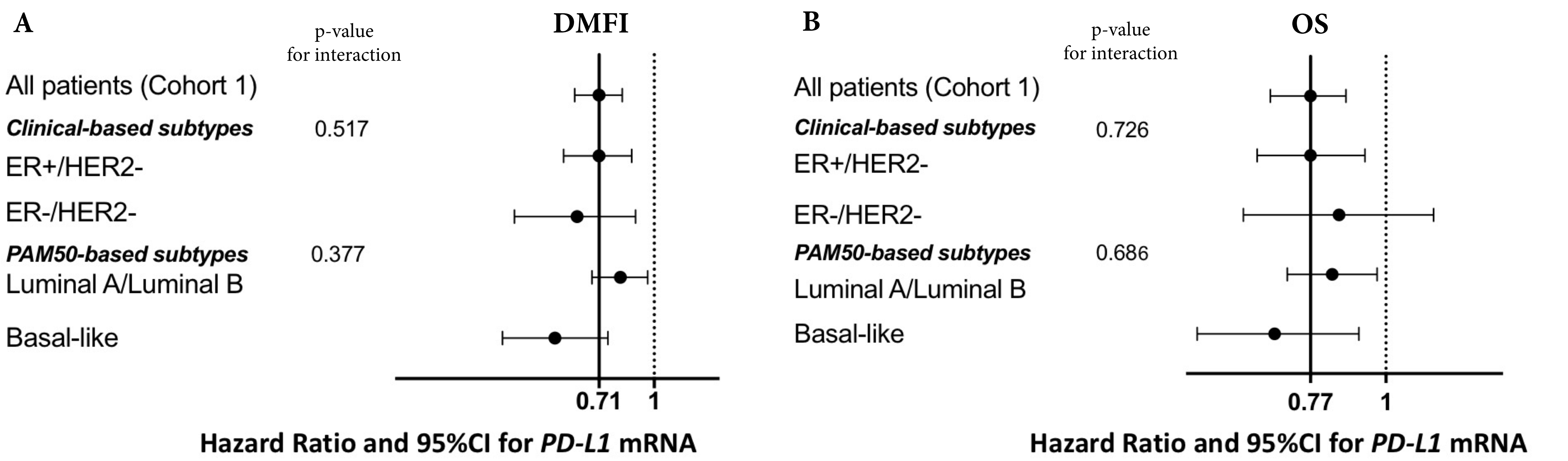

Supplement: Supplementary file 2 — Fig. S2. Prognostic value of PD‐L1 mRNA expression in cohort 1. Forest plots of HR for DMFI (A), and OS (B) both in the whole population and within clinical and PAM50‐based subtypes; HR is the relative hazard for a one‐standard deviation increase in the PD‐L1 mRNA expression. Cox regression multivariable models were adjusted for LN status and tumor size. [file MOL2-14-951-s002.pdf]
