# Supplementary material for: Programmed death‐ligand 1 gene expression is a prognostic marker in early breast cancer and provides additional prognostic value to 21‐gene and 70‐gene signatures in estrogen receptor‐positive disease
Source: Mol Oncol. 2020 Mar 20;14(5):951–63. doi: 10.1002/1878-0261.12654 (PMC7191187; doi:10.1002/1878-0261.12654)

Supplementary Figure S3. **Lymphocytic infiltration and its prognostic value in cohort 1. (A) Representative tissue areas immunohistochemically (IHC) stained for CD3 with low and high lymphocytic infiltration. Original magnification x400; (B) Survival analysis (Kaplan-Meier estimate) with the distant metastasis-free interval (DFMI) as a clinical endpoint in breast cancer patients spit by CD3 IHC expression**

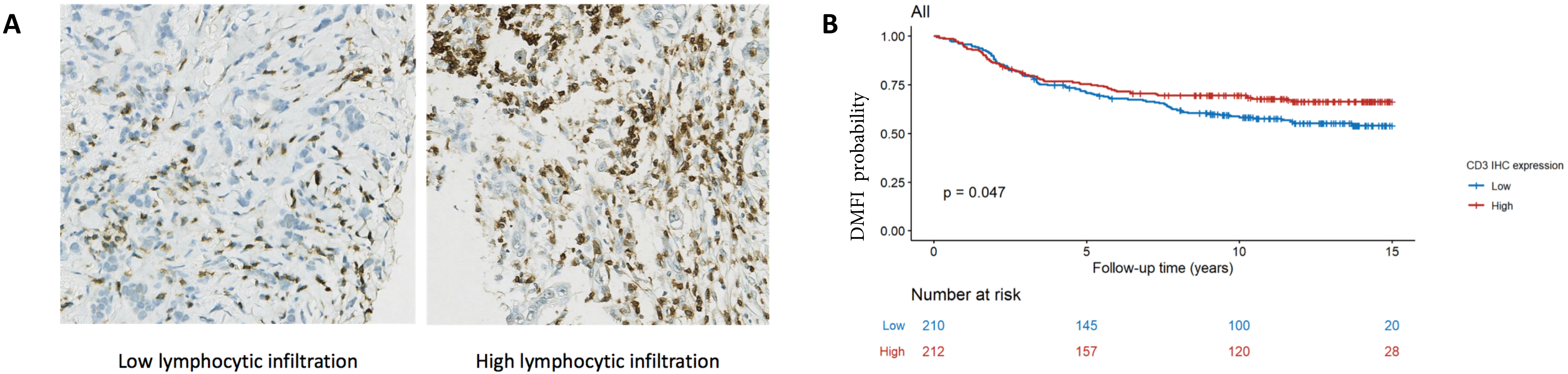

Supplement: Supplementary file 3 — Fig. S3. Lymphocytic infiltration and its prognostic value in cohort 1. (A) Representative tissue areas immunohistochemically (IHC) stained for CD3 with low and high lymphocytic infiltration. Original magnification x400; (B) Survival analysis (Kaplan‐Meier estimate) with the DMFI as a clinical endpoint in breast cancer patients spit by CD3 IHC expression. [file MOL2-14-951-s003.pdf]
