# Supplementary figures and images for: Programmed death‐ligand 1 gene expression is a prognostic marker in early breast cancer and provides additional prognostic value to 21‐gene and 70‐gene signatures in estrogen receptor‐positive disease
Source: Mol Oncol. 2020 Mar 20;14(5):951–63. doi: 10.1002/1878-0261.12654 (PMC7191187; doi:10.1002/1878-0261.12654)

## Supplementary Figure S4

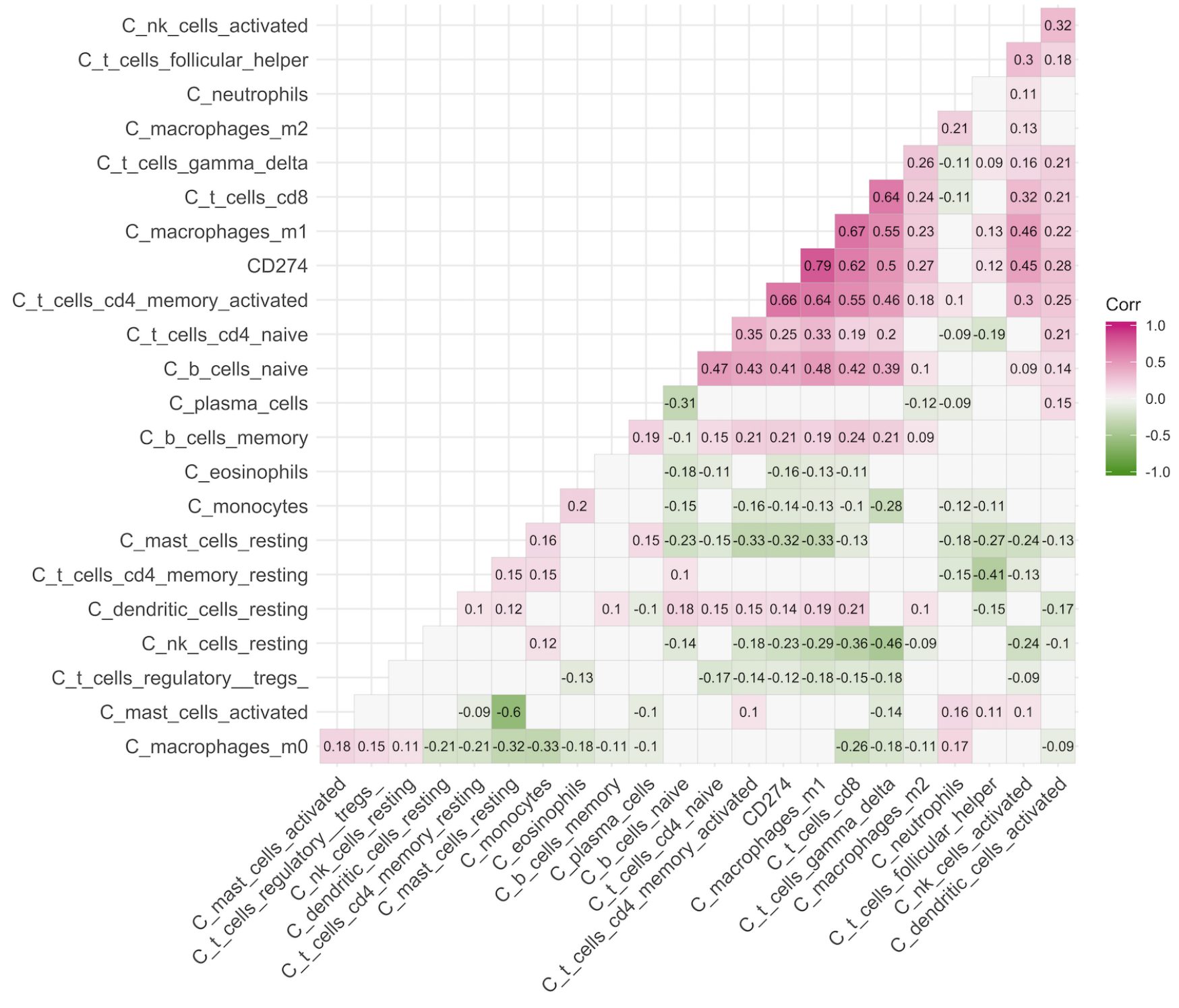

Supplement: Supplementary file 4 — Fig. S4. Correlation matrix depicting PD‐L1 transcript with different absolute fraction scores as derived from CIBERSORT‐based immune cell subpopulations in cohort 1. [file MOL2-14-951-s004.pdf]
