# Supplementary material for: Programmed death‐ligand 1 gene expression is a prognostic marker in early breast cancer and provides additional prognostic value to 21‐gene and 70‐gene signatures in estrogen receptor‐positive disease
Source: Mol Oncol. 2020 Mar 20;14(5):951–63. doi: 10.1002/1878-0261.12654 (PMC7191187; doi:10.1002/1878-0261.12654)
Supplement: Supplementary file 5 — Table S1. Patient characteristics for all patients and split by PD‐L1 total protein expression in cohort 1. Table S2. Patient characteristics for all patients and split by PD‐L1 mRNA (median) expression in cohort 2. Table S3. Univariate analysis of PD‐L1 IHC expression with survival outcomes in cohort 1. Table S4. Multivariable analysis of PD‐L1 IHC expression with survival outcomes in cohort 1. Table S5. Univariate and multivariable analyses of PD‐L1 mRNA expression with survival outcomes in both cohorts. Table S6. Patient characteristics for patients split by CD3 protein expression (median) in cohort 1. Table S7. Added prognostic value of PD‐L1 mRNA to GS (categorical) in ER+/HER2− patients in both cohorts. [file MOL2-14-951-s005.docx]

**Supporting information (Tables) to:**

**Programmed Death Ligand-1 gene expression is a prognostic marker and provides additional prognostic value to 21-gene and 70-gene signatures in early breast cancer**

Ioannis Zerdes^1^, Emmanouil G. Sifakis^1^, Alexios Matikas^1,2^, Sebastian Chrétien^1^, Nicholas P. Tobin^1^, Johan Hartman^1,3^, George Z. Rassidakis^1,3^, Jonas Bergh^1,2^, Theodoros Foukakis^1,2^

**Authors’ adresses:** ^1^Department of Oncology-Pathology, Karolinska Institutet Stockholm, Sweden; ^2^Breast Center, Theme Cancer, Karolinska University Hospital, Stockholm, Sweden; ^3^Department of Pathology and Cytology, Karolinska University Hospital, Stockholm, Sweden

**Number of Supplementary Tables:** 7

**Supplementary Table S1.** Patient characteristics for all patients and split by PD-L1 total protein expression in cohort 1

| **Clinical and pathological characteristics** | **All  n (%)** | **PD-L1 negative n (%)*** | **PD-L1 positive n (%)*** | **p-value** |
| --- | --- | --- | --- | --- |
| **Number of patients** | 490 | 369 | 121 |  |
| **ER status** |  |  |  |  |
| ER- | 132 (26.9) | 73 (55.3) | 59 (44.7) | **<0.0001** |
| ER+ | 346 (70.6) | 288 (83.2) | 58 (16.8) |  |
| Unknown | 12 (2.4) | 8 (66.7) | 4 (33.3) |  |
| **PR status** |  |  |  |  |
| PR- | 129 (26.3) | 77 (59.7) | 52 (40.3) | **<0.0001** |
| PR+ | 233 (47.6) | 194 (83.3) | 39 (16.7) |  |
| Unknown | 128 (26.1) | 98 (76.6) | 30 (23.4) |  |
| **HER2 status** |  |  |  |  |
| HER2- | 358 (73.1) | 273 (76.3) | 85 (23.7) | **0.044** |
| HER2+ | 81 (16.5) | 53 (65.4) | 28 (34.6) |  |
| Unknown | 51 (10.4) | 43 (84.3) | 8 (15.7) |  |
| **Elston-Ellis grade** |  |  |  |  |
| Grade I | 42 (8.6) | 40 (95.2) | 2 (4.8) | **<0.0001** |
| Grade II | 217 (44.3) | 187 (86.2) | 30 (13.8) |  |
| Grade III | 225 (45.9) | 137 (60.9) | 88 (39.1) |  |
| Unknown | 6 (1.2) | 5 (83.3) | 1 (16.7) |  |
| **Lymph node status** |  |  |  |  |
| Negative | 209 (42.7) | 154 (73.7) | 55 (26.3) | 0.4952 |
| Positive | 267 (54.5) | 204 (76.4) | 63 (23.6) |  |
| Unknown | 14 (2.9) | 11 (78.6) | 3 (21.4) |  |
| **Tumor size** |  |  |  |  |
| ≤20mm | 229 (46.7) | 178 (77.7) | 51 (22.3) | 0.282 |
| >20mm | 249 (50.8) | 183 (73.5) | 66 (26.5) |  |
| Unknown | 12 (2.4) | 8 (66.7) | 4 (33.3) |  |
| **Age** |  |  |  |  |
| ≤45 | 111 (22.7) | 86 (77.5) | 25 (22.5) | 0.631 |
| 45-55 | 123 (25.1) | 92 (74.8) | 31 (25.2) |  |
| >55 | 256 (52.2) | 191 (74.6) | 65 (25.4) |  |
| **IHC subtypes** |  |  |  |  |
| ER+/HER2- | 263 (53.7) | 217 (82.5) | 46 (17.5) | **<0.0001** |
| HER2+ | 81 (16.5) | 53 (65.4) | 28 (34.6) |  |
| ER-/HER2- | 86 (17.6) | 48 (55.8) | 38 (44.2) |  |
| Unknown | 60 (12.2) | 51 (85.0) | 9 (15.0) |  |
| **PAM50-based subtypes** |  |  |  |  |
| Luminal A | 218 (44.5) | 195 (89.4) | 23 (10.6) | **<0.0001** |
| Luminal B | 96 (19.6) | 73 (76.0) | 23 (24.0) |  |
| HER2-enriched | 51 (10.4) | 30 (58.8) | 21 (41.2) |  |
| Basal-like | 107 (21.8) | 59 (55.1) | 48 (44.9) |  |
| Normal-like | 16 (3.3) | 11 (68.8) | 5 (31.2) |  |
| Unknown | 2 (0.4) | 1 (50.0) | 1 (50.0) |  |
| **Treatment** |  |  |  |  |
| Endocrine treatment | 147 (30.0) | 128 (87.1) | 19 (12.9) | **<0.0001** |
| Chemotherapy | 139 (28.4) | 78 (56.1) | 61 (43.9) |  |
| ET/CT | 189 (38.6) | 152 (80.4) | 37 (19.6) |  |
| Other treatment | 14 (2.9) | 11 (78.6) | 3 (21.4) |  |
| Unknown | 1 (0.2) | 0 (0.0) | 1 (100.0) |  |

**Abbreviations:** PD-L1: programmed death ligand 1; IHC: immunohistochemistry; ER: estrogen receptor; PR: progesterone receptor; HER2: human epidermal growth factor receptor 2; ET: endocrine treatment; CT: Chemotherapy

***** Percentage (%) is calculated according to PD-L1 IHC expression (negative vs. positive group)

**Supplementary Table S2.** Patient characteristics for all patients and split by *PD-L1* mRNA (median) expression in cohort 2

| **Clinical and pathological characteristics** | **All**  **n (%)** | ***PD-L1* mRNA low n (%)*** | ***PD-L1* mRNA high n (%)*** | **p-value** |
| --- | --- | --- | --- | --- |
| **Number of patients** | 1081 | 541 | 540 |  |
| **ER status** |  |  |  |  |
| ER- | 237 (21.9) | 88 (37.1) | 149 (62.9) | **<0.0001** |
| ER+ | 794 (73.5) | 426 (53.7) | 368 (46.3) |  |
| Unknown | 50 (4.6) | 27 (54.0) | 23 (46.0) |  |
| **PR status** |  |  |  |  |
| PR- | 340 (31.5) | 147 (43.2) | 193 (56.8) | **0.0035** |
| PR+ | 688 (63.6) | 364 (52.9) | 324 (47.1) |  |
| Unknown | 53 (4.9) | 30 (56.6) | 23 (43.4) |  |
| **HER2 status** |  |  |  |  |
| HER2- | 763 (70.6) | 379 (49.7) | 384 (50.3) | 0.5085 |
| HER2+ | 179 (16.6) | 84 (46.9) | 95 (53.1) |  |
| Unknown | 139 (12.9) | 78 (56.1) | 61 (43.9) |  |
| **Lymph node status** |  |  |  |  |
| Negative | 511 (47.3) | 245 (47.9) | 266 (52.1) | 0.2296 |
| Positive | 550 (50.9) | 284 (51.6) | 266 (48.4) |  |
| Unknown | 20 (1.9) | 12 (60.0) | 8 (40.0) |  |
| **Tumor size** |  |  |  |  |
| ≤20mm | 278 (25.7) | 135 (48.6) | 143 (51.4) | 0.6406 |
| >20mm | 761 (70.4) | 382 (50.2) | 379 (49.8) |  |
| Unknown | 42 (3.9) | 24 (57.1) | 18 (42.9) |  |
| **Age** |  |  |  |  |
| ≤45 | 185 (17.1) | 89 (48.1) | 96 (51.9) | 0.8157 |
| 45-55 | 258 (23.9) | 128 (49.6) | 130 (50.4) |  |
| >55 | 637 (58.9) | 323 (50.7) | 314 (49.3) |  |
| Unknown | 1 (0.1) | 1 (100.0) | 0 (0.0) |  |
| **IHC subtypes** |  |  |  |  |
| ER+/HER2- | 590 (54.6) | 320 (54.2) | 270 (45.8) | **<0.0001** |
| HER2+ | 179 (16.6) | 84 (46.9) | 95 (53.1) |  |
| ER-/HER2- | 173 (16.0) | 59 (34.1) | 114 (65.9) |  |
| Unknown | 139 (12.9) | 78 (56.1) | 61 (43.9) |  |
| **PAM50-based subtypes** |  |  |  |  |
| Luminal A | 443 (41.0) | 250 (56.4) | 193 (43.6) | **<0.0001** |
| Luminal B | 244 (22.6) | 141 (57.8) | 103 (42.2) |  |
| HER2-enriched | 123 (11.4) | 46 (37.4) | 77 (62.6) |  |
| Basal-like | 201 (18.6) | 75 (37.3) | 126 (62.7) |  |
| Normal-like | 65 (6.0) | 29 (44.6) | 36 (55.4) |  |
| Unknown | 5 (0.5) | 0 (0.0) | 5 (100.0) |  |

**Abbreviations:** PD-L1: programmed death ligand 1; ER: estrogen receptor; PR: progesterone receptor; HER2: human epidermal growth factor receptor 2;

***** Percentage (%) is calculated according to PD-L1 mRNA expression (low vs. high group)

**Supplementary Table S3.** Univariate analysis of PD-L1 IHC expression with survival outcomes in cohort 1

| **PD-L1 IHC expression in total cells [HR (95% CI)]** | | | | | | | | | | |
| --- | --- | --- | --- | --- | --- | --- | --- | --- | --- | --- |
| **Cohorts (endpoint) / Subtypes and sample size (number of events)** | **All** | **n** | **ER+/HER2-** | **n** | **ER-/HER2-** | **n** | **Luminal A/B** | **n** | **Basal-like** | **n** |
| **Cohort 1 (DMFI)** | 0.58 ** (0.39-0.87) | 562 (215) | 0.35 ** (0.16-0.76) | 283  (103) | 0.40 ^ns^ (0.14-1.01) | 93 (25) | 0.76 ns (0.42-1.35) | 356  (136) | 0.42 ^ns^ (0.17-1.01) | 122  (35) |
| **Cohort 1 (OS)** | 0.75 ^ns^ (0.53-1.05) | 562  (259) | 0.47 * (0.25-0.88) | 283  (121) | 0.79 ^ns^ (0.38-1.66) | 93  (35) | 0.84 ^ns^ (0.50-1.40) | 356  (161) | 0.68 ^ns^ (0.35-1.33) | 122  (48) |

DMFI and OS were used as clinical endpoints both for all patients and molecular/intrinsic subtypes

**Abbreviations:** HR: hazard ratio; CI: Confidence Interval; ER: estrogen receptor; HER2: human epidermal growth factor receptor 2; OS: overall survival; PD-L1: programmed death ligand 1; IHC: immunohistochemistry; DMFI: distant metastasis-free interval; OS: overall survival

- Cox regression multivariable models were adjusted for lymph node status and tumor size
- Reference: PD-L1 negative
- *: p<0.05; ** : p<0.01; *** : p<0.001; ns: not significant
- n: number of patients per subgroup included in the univariate analysis and number of events
- Total cells: tumor or immune cells

**Supplementary Table S4.** Multivariable analysis of PD-L1 IHC expression with survival outcomes in cohort 1

| **PD-L1 IHC expression in total cells [HR (95% CI)]** | | | | | | | | | | |
| --- | --- | --- | --- | --- | --- | --- | --- | --- | --- | --- |
| **Cohorts (endpoint) / Subtypes and sample size (number of events)** | **All** | **n** | **ER+/HER2-** | **n** | **ER-/HER2-** | **n** | **Luminal A/B** | **n** | **Basal-like** | **n** |
| **Cohort 1 (DMFI)** | 0.52 ** (0.34-0.80) | 560 (213) | 0.35 ** (0.16-0.76) | 283 (103) | 0.24 * (0.07-0.76) | 93 (25) | 0.76 ns (0.45-1.36) | 356  (136) | 0.38 * (0.16-0.90) | 122  (35) |
| **Cohort 1 (OS)** | 0.66 * (0.46-0.94) | 560 (257) | 0.48 * (0.25-0.89) | 283  (121) | 0.63 ^ns^ (0.29-1.36) | 93  (35) | 0.85 ^ns^ (0.51-1.42) | 356  (161) | 0.63 ^ns^ (0.32-1.22) | 122  (48) |

DMFI and OS were used as clinical endpoints both for all patients and molecular/intrinsic subtypes

**Abbreviations:** HR: hazard ratio; CI: Confidence Interval; ER: estrogen receptor; HER2: human epidermal growth factor receptor 2; OS: overall survival; PD-L1: programmed death ligand 1; IHC: immunohistochemistry; DMFI: distant metastasis-free interval; OS: overall survival

- Cox regression multivariable models were adjusted for lymph node status and tumor size
- Reference: PD-L1 negative
- *: p<0.05; ** : p<0.01; *** : p<0.001; ns: not significant
- n: number of patients per subgroup included in the multivariable analysis and number of events
- Total cells: tumor or immune cells

**Supplementary Table S5.** Univariate and multivariable analyses of *PD-L1* mRNA expression with survival outcomes in both cohorts

|  | ***PD-L1* mRNA expression** | | | | | | | | | |
| --- | --- | --- | --- | --- | --- | --- | --- | --- | --- | --- |
|  | **Univariate analysis** | | | | | **Multivariable analysis** | | | | |
|  | **HR (95% CI)** | | |  |  | **HR (95% CI)** | | |  |  |
| **Cohorts (endpoint) / Subtypes** | **All** | **ER+/HER2-** | **ER-/HER2-** | **LA/LB** | **Basal-like** | **All** | **ER+/HER2-** | **ER-/HER2-** | **LA/LB** | **Basal-like** |
| **Cohort 1 (DMFi)** | 0.74 *** (0.64-0.85) | 0.71 *** (0.58-0.87) | 0.61 ** (0.42-0.88) | 0.80 * (0.68-0.95) | 0.54 *** (0.39-0.76) | 0.71 *** (0.61-0.82) | 0.71 ** (0.57-0.87) | 0.62 * (0.42-0.89) | 0.81 * (0.68-0.96) | 0.54 *** (0.39-0.75) |
| **Number of pts (number of events)** | 562  (215) | 283  (103) | 93  (25) | 356  (136) | 122  (35) | 560  (213) | 283  (103) | 93  (25) | 356  (136) | 122  (35) |
|  | | | | | | | | | | |
| **Cohort 2 (PFI)** | 0.78 ** (0.65-0.93) | 0.66 ** (0.50-0.88) | 0.64 * (0.44-0.95) | 0.81 ^ns^ (0.64-1.04) | 0.65 * (0.46-0.93) | 0.76 ** (0.64-0.91) | 0.67 ** (0.50-0.89) | 0.60 * (0.40-0.89) | 0.87 ^ns^ (0.68-1.12) | 0.63 * (0.44-0.91) |
| **Number of pts (number of events)** | 1080  (129) | 589  (52) | 173  (28) | 686  (69) | 201  (30) | 1075  (129) | 589  (52) | 173  (28) | 686  (69) | 201  (30) |
|  | | | | | | | | | | |
| **Cohort 1**  **(OS)** | 0.81 *** (0.72-0.92) | 0.77 ** (0.64-0.92) | 0.84 ^ns^ (0.60-1.17) | 0.82 * (0.71-0.96) | 0.70 * (0.52-0.93) | 0.77 *** (0.67-0.87) | 0.77 ** (0.64-0.93) | 0.85 ^ns^ (0.61-1.18) | 0.83 * (0.71-0.97) | 0.68 ** (0.52-0.91) |
| **Number of pts (number of events)** | 562  (259) | 283  (121) | 93  (35) | 356  (161) | 122 | 560  (257) | 283  (121) | 93  (35) | 356  (161) | 122  (48) |
|  | | | | | | | | | | |
| **Cohort 2**  **(OS)** | 0.86 ^ns^ (0.73-1.02) | 0.99 ^ns^ (0.76-1.28) | 0.61 ** (0.42-0.88) | 0.91 ^ns^ (0.73-1.14) | 0.65 * (0.46-0.94) | 0.85 ^ns^ (0.72-1.01) | 1.01 ^ns^  (0.77-1.32) | 0.56 ** (0.38-0.83) | 0.95 ^ns^ (0.76-1.19) | 0.64 * (0.45-0.92) |
| **Number of pts (number of events)** | 1080  (138) | 589  (53) | 173  (28) | 686  (80) | 201  (28) | 1075  (138) | 589  (53) | 173  (28) | 686  (80) | 201  (28) |

DMFI (cohort 1), PFI (cohort 2) and OS (both cohorts) were used as clinical endpoints both for all patients and for molecular/intrinsic subtypes.

**Abbreviations:** HR: hazard ratio; CI: Confidence Interval; ER: estrogen receptor; HER2: human epidermal growth factor receptor 2; OS: overall survival; PD-L1: programmed death ligand 1; IHC: immunohistochemistry; DMFI: distant metastasis-free interval; PFI: progression-free interval; OS: overall survival; LA/LB: Luminal A/Luminal B

- Cox regression multivariable models were adjusted for lymph node status and tumor size
- HR is the relative hazard for a one-standard deviation increase in the *PD-L1* mRNA expression
- *: p<0.05; ** : p<0.01; *** : p<0.001; ns: not significant
- n: number of patients per subgroup included in the univariate/multivariable analyses and number of events

**Supplementary Table S6.** Patient characteristics for patients split by CD3 protein expression (median) in cohort 1

| **Clinical and pathological characteristics** | **CD3 IHC low  n (%)*** | **CD3 IHC high  n (%)*** | **p-value** |
| --- | --- | --- | --- |
| **Number of patients** | 210 | 212 |  |
| **ER status** |  |  |  |
| ER- | 51 (41.8) | 71 (58.2) | **0.022** |
| ER+ | 156 (54.2) | 132 (45.8) |  |
| Unknown | 3 (25.0) | 9 (75.0) |  |
| **PR status** |  |  |  |
| PR- | 51 (42.1) | 70 (57.9) | **0.014** |
| PR+ | 110 (56.4) | 85 (43.6) |  |
| Unknown | 49 (46.2) | 57 (53.8) |  |
| **HER2 status** |  |  |  |
| HER2- | 156 (51.0) | 150 (49.0) | 0.182 |
| HER2+ | 35 (42.7) | 47 (57.3) |  |
| Unknown | 19 (55.9) | 15 (44.1) |  |
| **Elston-Ellis grade** |  |  |  |
| Grade I | 12 (40.0) | 18 (60.0) | 0.267 |
| Grade II | 101 (53.7) | 87 (46.3) |  |
| Grade III | 96 (47.8) | 105 (52.2) |  |
| Unknown | 1 (33.3) | 2 (66.7) |  |
| **Lymph node status** |  |  |  |
| Negative | 90 (52.6) | 81 (47,4) | 0.367 |
| Positive | 115 (48.1) | 124 (51.9) |  |
| Unknown | 5 (41.7) | 7 (58.3) |  |
| **Tumor size** |  |  |  |
| ≤20mm | 98 (51.0) | 94 (49.0) | 0.692 |
| >20mm | 108 (49.1) | 112 (50.9) |  |
| Unknown | 4 (40.0) | 6 (60.0) |  |
| **Age** |  |  |  |
| ≤45 | 56 (56.0) | 44 (44.0) | 0.286 |
| 45-55 | 56 (50.5) | 55 (49.5) |  |
| >55 | 98 (46.4) | 113 (53.6) |  |
| **IHC subtypes** |  |  |  |
| ER+/HER2- | 122 (55.7) | 97 (44.3) | **0.024** |
| HER2+ | 35 (42.7) | 47 (57.3) |  |
| ER-/HER2- | 32 (40.5) | 47 (59.5) |  |
| Unknown | 21 (50.0) | 21 (50.0) |  |
| **PAM50-based subtypes** |  |  |  |
| Luminal A | 103 (56.6) | 79 (43.4) | **0.037** |
| Luminal B | 44 (53.0) | 39 (47.0) |  |
| HER2-enriched | 21 (42.0) | 29 (58.0) |  |
| Basal-like | 37 (38.9) | 58 (61.1) |  |
| Normal-like | 4 (36.4) | 7 (63.6) |  |
| Unknown | 1 (100.0) | 0 (0.0) |  |
| **Treatment** |  |  |  |
| Endocrine treatment | 71 (60.2) | 47 (39.8) | **0.041** |
| Chemotherapy | 55 (42.3) | 75 (57.7) |  |
| ET/CT | 78 (48.1) | 84 (51.9) |  |
| Other treatment | 6 (54.5) | 5 (45.5) |  |
| Unknown | 0 (0.0) | 1 (100.0) |  |

**Abbreviations:** CD3: cluster of differentiation 3; IHC: immunohistochemistry; ER: estrogen receptor; PR: progesterone receptor; HER2: human epidermal growth factor receptor 2; ET: endocrine treatment; CT: Chemotherapy

***** Percentage (%) is calculated according to CD3 IHC expression (low vs. high group)

**Supplementary Table S7.** Added prognostic value of *PD-L1* mRNA to gene-signatures (categorical) in ER+/HER2- patients in both cohorts

|  |  | **Cohort 1  (n = 283)** | | | | **Cohort 2  (n = 590)** | | | |
| --- | --- | --- | --- | --- | --- | --- | --- | --- | --- |
|  |  |  |  |  |  |  |  |  |  |
|  |  | **LR-Δχ^2^** | **p-value** | **LR-Δχ^2^** | **p-value** | **LR-Δχ^2^** | **p-value** | **LR-Δχ^2^** | **p-value** |
|  |  | **DMFI** | | **OS** | | **PFI** | | **OS** | |
| **21-gene** | | | | | | | | | |
| 21-gene + *PD-L1* vs 21-gene | | 11.607 | 0.001 | 7.963 | 0.005 | 8.317 | 0.004 | 0.002 | 0.962 |
| **70-gene** | |  |  |  |  |  |  |  |  |
| 70-gene + *PD-L1* vs 70-gene | | 13.252 | <0.001 | 8.945 | 0.003 | 7.956 | 0.005 | 0.031 | 0.860 |
|  |  |  |  |  |  |  |  |  |  |
|  |  | **C-index** | | | | | | | |
|  |  | **DMFI** | | **OS** | | **PFI** | | **OS** | |
| **21-gene** | |  |  |  |  |  |  |  |  |
| 21-gene | | 0.598 | | 0.565 | | 0.606 | | 0.556 | |
| 21-gene + *PD-L1* | | 0.661 | | 0.612 | | 0.663 | | 0.560 | |
| **70-gene** | |  |  |  |  |  |  |  |  |
| 70-gene | | 0.579 | | 0.557 | | 0.546 | | 0.540 | |
| 70-gene + *PD-L1* | | 0.644 | | 0.605 | | 0.637 | | 0.537 | |

Gene expression signatures were evaluated as categorical variables

**Abbreviations:** PD-L1: programmed death ligand 1; LR: likelihood ratio; C-index: concordance index; DMFI: distant metastasis-free interval; PFI: progression-free interval; OS: overall survival
